# Identification and anti-bacterial property of endophytic actinobacteria from *Thymes kotschyanus*, *Allium hooshidaryae*, and *Cerasus microcarpa*

**DOI:** 10.1038/s41598-023-40478-x

**Published:** 2023-08-12

**Authors:** Yaser Delbari, Yaser Mohassel, Elham Kakaei, Yadollah Bahrami

**Affiliations:** 1 Department of Medical Biotechnology, School of Medicine, Kermanshah University of Medical Sciences, Kermanshah, Iran; 2 Department of Clinical Biochemistry, School of Medicine, Kermanshah University of Medical Sciences, Kermanshah, Iran; 3 Medical Biology Research Center, Kermanshah University of Medical Sciences, Kermanshah, Iran; 4 Department of Medical Biotechnology, School of Medicine, College of Medicine and Public Health, Flinders University, Adelaide, SA 5042 Australia; 5 Advanced Marine Biomanufacturing Laboratory, Centre for Marine Bioproducts Development, College of Medicine and Public Health, Flinders University, Adelaide, SA 5042 Australia

**Keywords:** Biological techniques, Biotechnology, Microbiology, Plant sciences, Diseases, Medical research

## Abstract

The arbitrary and overuses of antibiotics have resulted in the emergence of multidrug resistance bacteria which encounters human to a serious public health problem. Thus, there is an ever-increasing demand for discovery of novel effective antibiotics with new modes of function against resistant pathogens. Endophytic actinobacteria (EA) have currently been considered as one of the most prospective group of microorganisms for discovery of therapeutic agents. This study aimed to isolate EA from *Thymes kotschyanus*, *Allium hooshidaryae*, and *Cerasus microcarpa* plants and to evaluate their antibacterial properties. The healthy samples were collected, dissected and surface-sterilized before cultured on four different selection media at 28 °C. Nine EA were isolated and identified based on morphological and molecular properties, and scanning electron micrograph analyses. Based on phylogenetic analysis, they were taxonomically grouped into four families *Streptomycetaceae, Nocardiaceae, Micromonosporaceae*, and *Pseudonocardiaceae*. Their branched aerial mycelia produced chains of cylindrical or cube or oval shaped spores with smooth or rough surfaces. Four strains; IKBG03, IKBG05, IKBG13, and IKBG17 had less than 98.65% sequence similarity to their closely related strains, which constitute them as novel species/strains. Besides, three strains; IKBG05, IKBG13, and IKBG18 were reported as endophytes for the first time. Preliminary antibacterial activity conducted on the all isolates revealed potent antibacterial effects against *Staphylococcus aureus*, *Escherichia coli*, and *Pseudomonas aeruginosa*. All isolates strongly inhibited the growth of at least one of the tested pathogens. Our results reveals that the test plants are novel sources for isolating a diverse group of rare and common actinobacteria that could produce a wide range of novel biologically active natural products with antibacterial activity which have a great potential in pharmaceutical and biotechnological applications.

## Introduction

Antibiotics are among the most widely used drugs in the world^1^. This, in addition to genomic mutations, translocations, and genetic alterations, has led to the development of microbial resistance to existing antibiotics. As a result, millions of people around the world, mostly children and the elderly, die each year due to increased bacterial resistance^2^. Due to this fact, it is indispensable to discover and develop novel and more powerful bioactive compounds and antibiotics from unexplored environments towards drugs resistant bacteria. Despite the efforts, we are still facing an increasing demand for new effective antibiotics, since microbial warfare seems to be inevitable in the near future^3^. Therefore, scientists have sought to identify and introduce more effective antibiotics over the past three decades^4^. In recent years, untapped drug sources have attracted a great deal of attention among researchers. Hence, many methods and technologies have been studied and conducted to discover new drugs^5^.

One of these attractive sources is microorganisms from unknown environmental origin. Natural products have been used for millennia to treat different infections. They provide us a great opportunity for discovery of therapeutic agents. The natural products of microorganisms are relatively cost-effective, affordable and justified for a variety of economic and non-economic reasons including non-toxic to host and active in low concentration, diversity in their environments, genetic potential, and cheap industrial production. Search for microorganisms in lesser-known environments such as mountainous, marine, and desert environments as well as those which coexist with medicinal plants which have been less studied due to difficulties in sampling, transport, storage, etc., can be a great reservoir for discovery new therapeutic compounds. Within microorganisms, the family of actinomycetes, especially the genus *Streptomyces*, still is the biggest producer of secondary metabolites and bioactive compounds^6^, accounting for over 70% of commercially useful antibiotics^7^. In addition, many bioactive substances with anti-tumor, anti-fungal, anti-inflammatory, and anti-cancer properties have been reported from them, which introduces these microorganisms as one of the promising sources for the production of new drugs^8–10^. Microbes commonly coexist in diverse communities in nature. Endophytic actinobacteria that coexist with medicinal plants residing within their robust tissues have very beneficial and important effects on the survival and life of their hosts, which are mostly unknown^11,12^. Plants harbor novel and diverse range of actinobacteria, and have always been considered as one of the new untapped sources for isolation of EA. Isolation of actinobacteria strains from different environments will probably lead to the identification of new species with high ability to produce bioactive compounds^13^. Accordingly, the isolation and identification of actinobacteria have recently become a productive area of research that has consequently led to the identification of novel *Actinomycete* species that need to be exploited to unveil possible biosynthetic pathways and discover new bioactive natural metabolites.

Actinobacteria as one of the largest ubiquitous bacterial phylum are generally Gram-positive, having high percentage of G + C bases in their genome, mycelial growth, and spore production. They are widely distributed in both normal and extreme aquatic and terrestrial ecosystems^6,14–17^. Most of them are free-living microorganisms which use the remains of other organisms as saprophytes and also spend most of their lives as spores, especially in harsh environmental conditions^18,19^. Actinobacteria are prolific producers of bioactive metabolites with a diverse range of chemically structure complexity^20^. The actinobacteria phylum currently includes the most prospective group of microorganisms for the discovery of biologically active compounds including peptides, polyketides, macrolides, quinolones^21^. Actinobacteria alone produce approximately two-thirds of all know antibiotics in the market with a diverse range of biological activities namely cytotoxic, antiparasitic, antibacterial, anticancer or antiproliferative, antifungal, antimicrobial, insecticidal and immunosuppressive activities^6^. Actinobacteria also play an important role in the recycling and degradation organic substances from dead organisms to a depth of two meters in the soil^22,23^. They can indirectly inhibit the growth of plant pathogens through biological control or antagonistic activities, especially the production of extracellular enzymes or antibiotics^24^, or produce compounds, which directly promote plant growth and facilitate the absorption of soil nutrients, such as nitrogen fixation, phytohormones and siderophores production, dissolution of soil minerals, etc.^11,25^. Due to the climatic and geographical diversity of Iran, a broad spectrum of plants and subsequently endophytic microorganisms are available.

Having over 8,000 plants species, the Iranian ecosystem has a very high potential to become a source of unknown endophytic actinobacteria (EA) with unique metabolic profile and properties which can be widely used for discovery and development of new drugs and even genetic engineering of useful strains to improve their properties. To date, however, very little is known about the EA of the native plants in Iran. Therefore, the exploration of potential actinobacteria from untapped habitats is a step-wise approach for discovery of novel antibiotics to meet the current needs to cope with the life-threatening infections. *Allium hooshidaryae, Cerasus microcarpa,* and *Thymes kotschyanus* Boiss & Hohen plants have generally been consumed as traditional medicine for many years due to their medicinal properties. Modern pharmacology of *Allium hooshidaryae, Cerasus microcarpa, and Thymes kotschyanus* indicated that the essential oils of these plants have anticancer, anti-anti-inflammatory properties. *Allium hooshidaryae* is a native wild plant in northwestern Iran, traditionally used as folk medicines. However, so far, no study has been published on their microbial diversity and endophytic populations. Therefore, the aims of this study were to isolate and phylogenetically identify EA from abovementioned plants and to evaluate the antibacterial effects of their extracts against *Staphylococcus aureus* ATCC 25923, *Escherichia coli* ATCC 25922, and *Pseudomonas aeruginosa* ATCC 27853.

## Results

### Assessment of surface sterilization

Surface sterilization is a crucial step for the isolation of plant endophytes. The final rinse water of surface-sterilized was cultured on ISP2 medium, and no microbial colony was observed after two weeks of incubation at 28 °C which highlights the effectiveness and success of the surface-sterilization protocol in suppressing the growth of unwanted phyllospheric microorganisms.

### Isolation of endophytic actinobacteria

**Figure 1:**
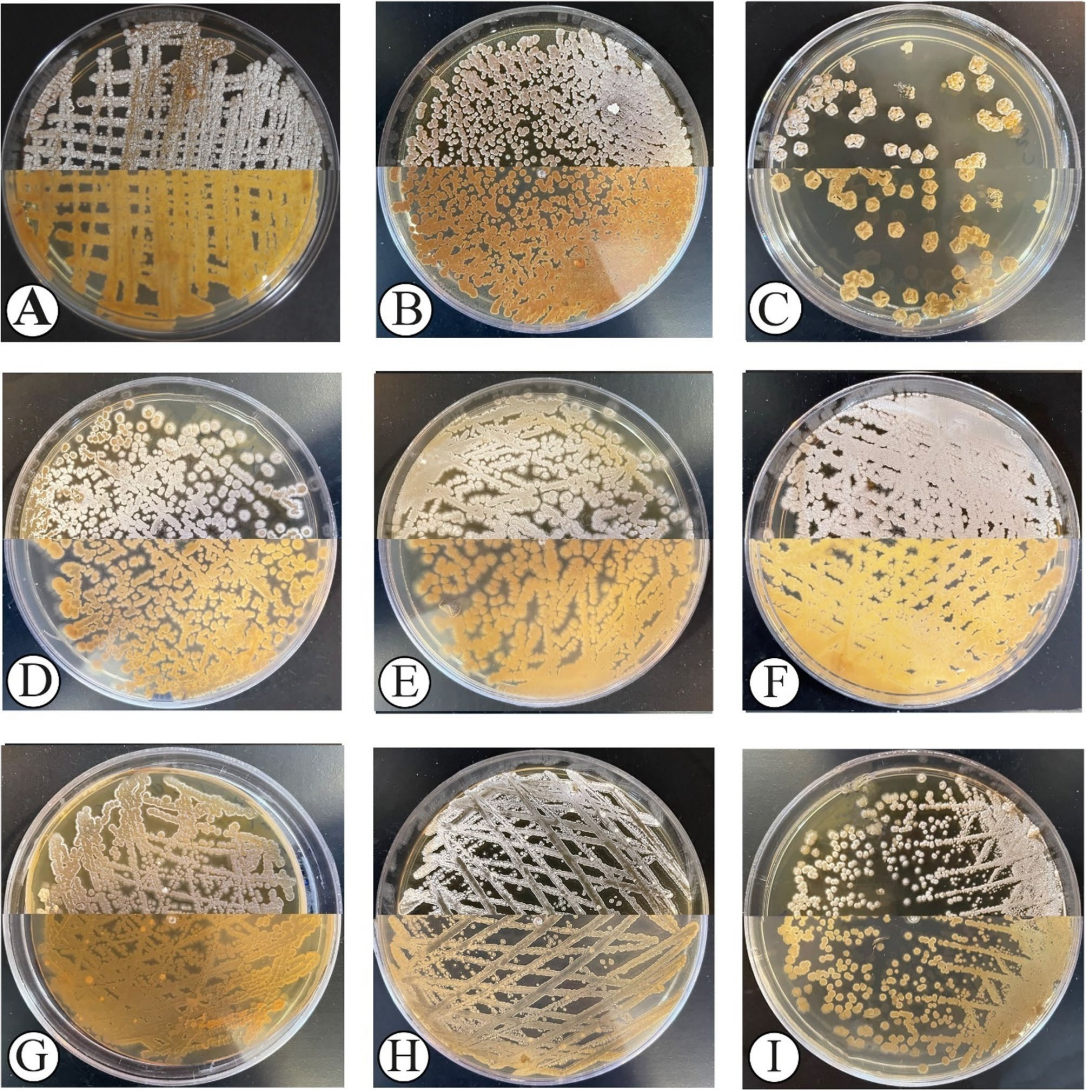
Morphological appearance of actinobacteria strains after two weeks of incubation onto ISP2 at 28°C. IKBG03 (**A**), IKBG05 (**B**), IKBG07 (**C**), IKBG13 (**D**), IKBG14 (**E**), IKBG17 (**F**), IKBG18 (**G**), IKBG19 (**H**), IKBG20 (**I**). The upper half of each plate shows the front of culture, while the lower half shows the back of each culture.

**Figure 2:**
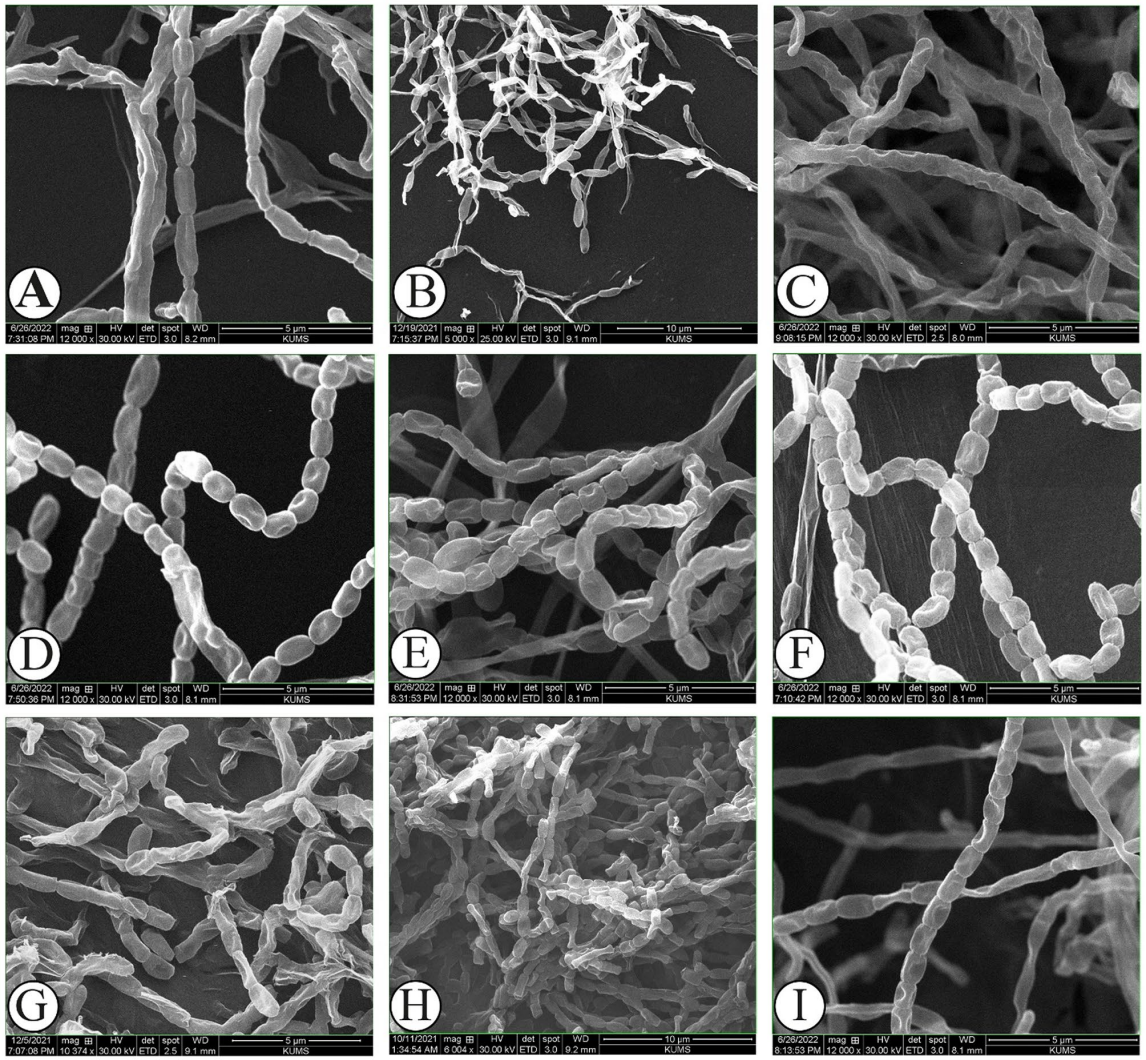
Scanning electron micrograph of aerial mycelia and spore chain ornamentation of actinobacteria isolates. The images show SEM analyses of mycelia and spore chain morphology and arrangements of strain from a 10-day old culture grown on ISP2 at 28 ºC. IKBG03 (**A**) (straight branched mycelia and spore chains with large cylindrical indent surface spores), IKBG05 isolate (**B**) (straight-branched mycelia and spore chains with large sausage- shaped, and indent surface spores), IKBG07 (**C**) (straight-branched mycelia and spore chains with cylindrical shaped, and indent surface spores), IKBG13 (**D**) (straight-branched mycelia with twisted end, and spore chains with small oval-shaped, and indent surface spores), IKBG14 (**E**) (straight-highly branched mycelia at the end and spore chains with cubed- shape, and indent smooth surface spores), IKBG17 (**F**) (spiral mycelia and spore chains with small cubed-shape spores, and indent smooth surface spores), IKBG18 (**G**) spiral mycelia and spore chains with cylindrical-shape spores, and indent coarse surface spores, IKBG19 (**H**) straight mycelia and spore chains with cubed- shape spores, and indent smooth surface spores, IKBG20 (**I**) straight-branched mycelia and spore chains with small oval- shaped, and indent surface spores. Scanning electron micrograph showing areal mycelia differentiate into straight to spiral spore chains with cylindrical or cubed or ellipsoid shaped spores.

To isolate as many actinobacteria as possible, the samples were plated simultaneously on four different media including TWYE, YECD, PDA and HVA. A total of nine different isolates recovered from different tissues of *Thymes kotschyanus*, *Cerasus microcarpa*, and *Allium hooshidaryae* on the media after 4–8 weeks of incubation at 28 ºC. The morphological isolation and purification of colonies were conducted onto ISP2 in order to identify the isolates. ISP2 was also used as a master plate to store colonies for further analyses. The isolates were then characterized based on their molecular properties. Most isolates had different pigmentation as demonstrated in Fig. 1. The color of colonies varied as summarized in Table 1. The strains produced an abundant white to orange yellow, light yellow, pale orange yellow, light orange yellow, greenish white, beige or brownish or light greenish-gray straight aerial mycelia, and appeared in recti flexible, occasionally twisted, which differentiate into straight or spiral spore chains after 10–14 days of culture on ISP2 medium (Supplementary Fig. 1 and Supplementary Fig. 2). The substrate mycelia of strains were generally lengthy. The morphology of mycelia and spores as well as spore chain ornamentations of the isolates were also examined using light and scanning electron microscopes (SEM). The scanning electron micrograph of the isolates are shown in Fig. 2. The spores had cylindrical or cubed or oval-shaped with smooth or rough surfaces.

**Table 1:** Morphological characteristics of nine endophytic actinobacteria isolates from *Thymes kotschyanus*, *Allium hooshidaryae*, and *Cerasus microcarpa.*

Isolate	Color characterization		Microscopic characterization			Identified genus
	Aerial mycelium	Substrate mycelium	Mycelia	Spore chain	Spore surface/shape	
IKBG03	Greenish white	Brownish-yellow	Straight Extensively branched and rod-shaped fragments	Straight branched long chain of spores	Smooth-indent surface/ large cylindrical shaped spores	*Pseudonocardiaceae*
IKBG05	White	Brownish- yellow	Straight Extensively branched and rod-shaped fragments	Long chain of spores	Smooth-indent surface /Large sausage- shaped spores	*Pseudonocardiaceae*
IKBG07	White	Light olive brown	Straight Extensively branched	long chains of spores	Smooth-indent surface/ cylindrical shaped spores	*Streptomycetaceae*
IKBG13	Orange yellow	Yellowish-white	Straight Extensively branched and rod-shaped fragments with twisted-Flexuous ends	Long chains of spores (spore with ≠ 1 μm length)/ Straight to Flexuous	Smooth-indent surface/ small oval- shaped, spores	*Nocardiaceae*
IKBG14	Light yellow	Brilliant yellow	Straight Extensively branched and rod-shaped fragments and spiral ends	Long chains of spores /Straight to Flexuous	Coarse indent surface/cubed-shaped spores	*Nocardiaceae*
IKBG17	Brilliant orange yellow	Brilliant orange yellow	spiral Extensively branched and rod-shapes	Long Flexuous spore chains	Smooth and indent surface spores/Small cubed-shape,	*Streptomycetaceae*
IKBG18	Brilliant orange yellow	Orange yellow	Spiral Extensively branched and rod-shaped fragments	Spial spore chains	Rough to warty surface/ cylindrical-vibrio shape spores	*Micromonosporaceae*
IKBG19	Greenish white	Pale greenish yellow	Straight Extensively branched and rod-shapes	Straight and long chains of spores	Smooth indent surface/cubed-shape spores	*Streptomycetaceae*
IKBG20	Light greenish gray	Deep brown	straight-branched and rod-shaped mycelia	straight and long chains of spores	Smooth indent surface/small oval-shaped spores	*Streptomycetaceae*

The color scheme was based on the ISCC-NBS system.

Differential morphological and cultural characteristics include spore arrangement, spore surface ornamentation, growth conditions and the colors of the aerial and substrate mycelia on ISP2 medium are summarized in Table 1.

The isolates were distributed into four families *Streptomycetaceae, Nocardiaceae, Micromonosporaceae*, and *Pseudonocardiaceae* within class actinobacteria (Table 2), which is in agreement with previous reports^26^. Four isolates were obtained from roots, three isolates from stems, and two isolates from leaves of the examined plants. Over 50% of isolates were acquired from *Thymes kotschyanus* (five isolates), followed by *Cerasus microcarpa* (two strains), and *Allium hooshidaryae* (two strains). Among nine isolates, four were isolated from TWYE, two isolates from YECD and three from HVA culture media. The result indicated that TWYE was the suitable medium for the isolation, and yielded the highest number of isolates which all belonged to *Streptomyces* genera. Over 50% of isolates were found to be a member of rare actinobacteria. It was documented that the incubation time seems to be more influential than the ingredients of isolation media in yielding rare genera of actinobacteria^27^.

**Table 2:** molecular identification of nine EA isolated from *Thymes kotschyanus*, *Allium hooshidaryae*, and *Cerasus microcarpa* plants based on 16S rDNA analysis. The table summarizes the GenBank accession numbers of the strain sequences, similarities between strains and their closely related species/ strains as well as plants and tissue of origin, and families which belong to *Streptomycetacea*e, *Nocardiaceae*, *Micromonosporaceae*, and *Pseudonocardiaceae*.

Isolates	Closest homologs	Family	Pairwise similarity (%)	Origin plant	Tissue	Isolation medium	Accession no
IKBG03	*Umezawaea endophytica*	*Pseudonocardiaceae*	97.93	*Cerasus microcarpa*	Stem	YECD	MZ733426
IKBG05	*U. tangerina*	*Pseudonocardiaceae*	98.38	*Cerasus microcarpa*	Stem	YECD	MZ733428
IKBG07	*S. californicus*	*Streptomycetaceae*	99.01	*Thymes kotschyanus*	Root	TWYE	MZ733430
IKBG13	*Nocardia mangyaensis*	*Nocardiaceae*	98.61	*Allium hooshidaryae*	Stem	HVA	MZ733436
IKBG14	*Nocardia fluminea*	*Nocardiaceae*	98.66	*Thymes kotschyanus*	Root	HVA	MZ733440
IKBG17	*S. tendae*	*Streptomycetaceae*	98.12	*Thymes kotschyanus*	Leaf	TWYE	MZ733437
IKBG18	*Catellatospora sichuanensis*	*Micromonosporaceae*	99.65	*Allium hooshidaryae*	Leaf	HVA	MZ733441
IKBG19	*S. cyaneofuscatus*	*Streptomycetaceae*	99.93	*Thymes kotschyanus*	Root	TWYE	MZ580433
IKBG20	*S. badius*	*Streptomycetaceae*	99.51	*Thymes kotschyanus*	Root	TWYE	MZ820169

The isolates were different in mycelium color, pigmentation and sporulation. Most of isolates demonstrated strain specific characteristics on the ISP2 medium (Fig. 1). The color of the mycelia and the diffusible pigment were recorded after two weeks of incubation at 28 ºC. The aerial mycelium shaped monopodially branched spore-bearing hyphae with the shape of straight or loops, open or compact spirals with 2–4 curves. The isolates from *Cerasus microcarpa* were of stem samples, whereas the highest number of isolates from *Thymes kotschyanus* was from roots. The isolates of *Allium hooshidaryae* were from stems and leaves. All isolates showed a good sporulation after 10 days of incubation on ISP2, except IKBG18 which had poor sporulation even after 14 days (Fig. 2). The isolates formed well developed, branched substrate and aerial mycelia in which the latter differentiates into straight to spiral spore chains which consist of cylindrical or cubed or ellipsoid spores. The spores of strains had generally smooth-surfaces. Strain IKBG18 produced spiral chains of rough spores, while most of strains formed straight to flexuous chains of smooth surface spores (Table 1). IKBG13 and IKBG14 demonstrated straight to flexuous chains of smooth spores.

The mycelium structures were studied using SEM. The results revealed that the aerial mycelia differentiae into straight to spiral spore chains that were highly branched (Fig. 2). Scanning electron micrograph of strains grown on ISP2 medium exhibited that the sporophores are rectus flexibilis and the spores had smooth surfaces except IKBG18. IKBG13 and IKBG14 strains produced straight to flexuous or spiral spore chains on ISP2 medium.

### Molecular identification of isolates

**Figure 3:**
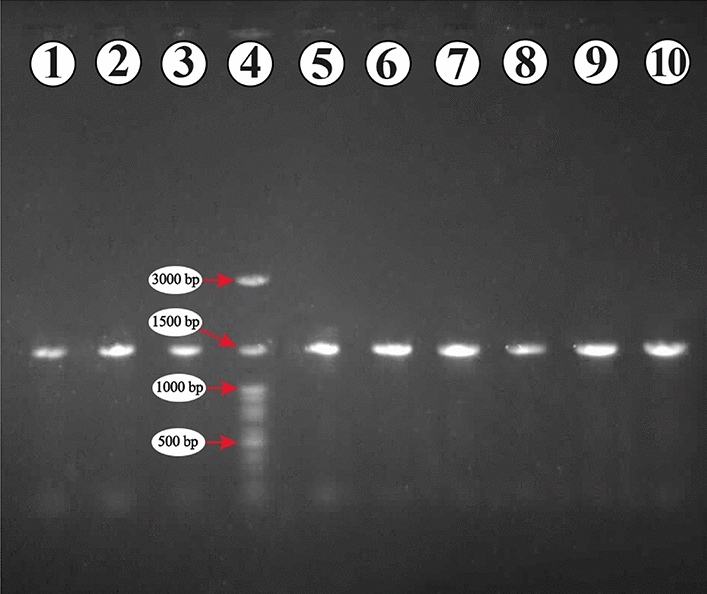
Gel electrophoresis of PCR products. PCR products of 16S rDNA of isolated strains using 27F and 1492R primers that expected to amplify a 1.5 kb product from strains. The 1.5 Kb amplified 16S rDNA fragments observed on 1% agarose gel electrophoresis. From left to right; IKBG03 (lane 1), IKBG05 (lane 2), IKBG07 (lane 3), DNA ladder, the major bands are labeled (lane 4, SinaClon, Iran), IKBG13 (lane 5), IKBG14 (lane 6), IKBG17 (lane 7), IKBG18 (lane 8), IKBG19 (lane 9), IKBG20 (lane 10).

The genomic DNA was extracted and amplified by PCR. The PCR products were run into gel electrophoresis as illustrates in Fig. 3. The original image of PCR gel electrophoresis is displayed in Supplementary Fig. 3.

The diversity of EA was analyzed using 16S rDNA sequencing data. The 16S rDNA sequence of the isolates were compared with the sequences in GenBank, NCBI database using BLASTn and aligned with the sequences retrieved from the NCBI GenBank and EzBioCloud database using the Clustal *W* method and trimmed manually where necessary in order to reveal the possible phylogenetic relationships between these sequences and those sequences of bacteria available in databank.

**Figure 4:**
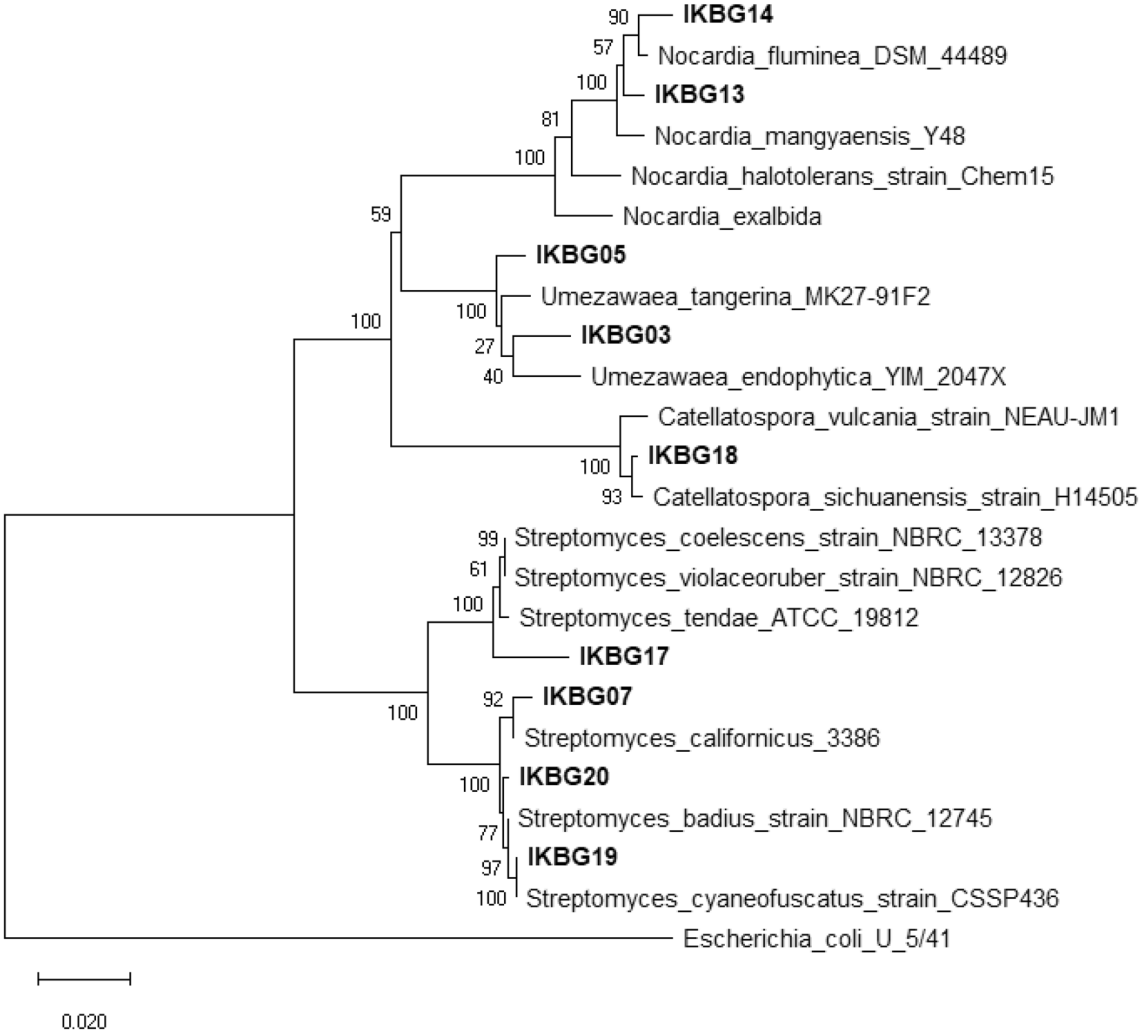
Phylogenetic tree of endophytic actinobacteria isolated from *Thymes kotschyanus, Allium hooshidaryae*, and *Cerasus microcarpa,* obtained by Neighbor–Joining method analysis representing phylogenetic relationships maximum likelihood method and their closely related type strains. Neighbor-joining tree presenting the phylogenetic position of the strains and their related species based on 16S rRNA gene sequences. The numbers at branch nodes are bootstrap values (%) from 1000 replicates, and scale bar is equal to 2 substitutions/100 nucleotides of 16S rDNA gene. *E. coli* U5/41 was used as outgroup. The bar scale, 0.02, represents the nucleotide substitutions per site/position. The numbers at branch nodes indicate bootstrap percentages derived from 1000 replications.

The phylogenetic analysis based on the 16S rDNA sequences revealed that the isolates belonged to four families *Streptomycetaceae*, *Nocardiaceae*, *Micromonosporaceae*, and *Pseudonocardiaceae* in which some were placed in rare actinobacteria and corroborated they are all actinobacteria (Table 2). In addition, all the 16S rDNA gene sequences data have been submitted to the GenBank and their corresponding accession numbers are listed in Table 2. The phylogenetic tree of the isolates was constructed based on neighbor joining tree method with their closely related strains as illustrated in Fig. 4. The alignment of their nucleotide’s sequences showed some similarities and differences. Neighbor-joining tree based on 16S rRNA gene sequences representing the phylogenetic relationships among the strains and other closely related species of actinobacteria. The nucleotide BLAST analysis showed 97.93 to 99.93% sequence similarity to their closest relative strains. The similarity of nucleotides in some strains such as IKBG19 and IKBG20 was as high as 99.93 and 99.51 to the data of 16S rDNA genes that are available in GenBank, respectively. The similarity of the 16S rDNA sequences and their closely related strains in the EzBioCloud database ranged from 97.93 to 99.93%. Of these, four isolates had less than 98.65% similarity with their closest related strains, which constituted them as novel species/ strains. The sequence of IKBG14 strain had also 98.66% similarity to its closest relative strain of *Nocardia fluminea*, which could also be assigned as a new species of Nocardia. BLASTn analysis of the 16S rRNA gene sequence revealed that IKBG07, IKBG18, IKBG19 and IKBG20 strains exhibited the highest similarities with *S. californicus*, *Catellatospora sichuanensis*, *S. cyaneofuscatus*, *S. badius*, respectively, and they had over 90% sequence similarity.

As the Fig. 4 indicates some of strains are *Nocardia* and some of those are *Streptomyces* strains. Within the isolates, *Streptomyces* (4 isolates) was prominent genus. Phylogenetic analysis revealed that the IKBG13 and IKBG14 formed a distinct clade with some strains of the genus *Nocardia*. However, both strains demonstrated distinct clade against KBG07, IKBG19 and IKBG20 strains that were most closely associated with the genus *Streptomyces*, isolated from the roots of *Thymes kotschyanus*. The strains IKBG07, IKBG17, IKBG19 and IKBG20 were found to form a unique clade that were different from other closely related strains, while IKBG03 and IKBG05 were placed in another distinct clade. The results of phylogenetic analysis also indicated that strain IKBG19 is associated with *Streptomyces cyaneofuscatus* under a bootstrap supported value of %100.

### Antibacterial activity of endophytic actinobacteria

The antibacterial properties of isolates were screened against three different strains namely *Staphylococcus aureus* ATCC 25923, *Escherichia coli* ATCC 25922, and *Pseudomonas aeruginosa* ATCC 27853. Preliminary data revealed that all nine isolates had strong inhibitory activity against at least one pathogenic bacterium. Some of the isolates exhibited a wide spectrum of inhibitory activity against target bacteria ranging from two to all of the test organisms. IKBG19 had inhibitory effects against all target strains, while IKBG05 exhibited the weakest antibacterial activity, which was effective only against one pathogen. The inhibitory effect of isolates against *P. aeruginosa* was the most frequent detected antimicrobial activity. The rare identified actinobacteria (IKBG03, IKBG05, IKBG13, IKBG18, IKBG14) also showed antagonist activity against test bacteria. In some cases, the activity of tested extracts was comparatively greater than positive control.

Overall, crude methanolic extracts had greater potential antibacterial activities against the test pathogens than ethyl acetate extracts. The highest antibacterial effect of both extracts was on *P. aeruginosa*. Generally, the methanolic extracts of the isolates displayed stronger antibacterial properties against *S. aureus* than their ethyl acetate extracts. IKBG03, IKBG7, IKBG13, IKBG14, IKBG18, IKBG17, IKBG19, and IKBG20 had antibacterial activity against *S. aureus*. Most of strains had no effect on *E*. *coli,* except methanolic extracts of IKBG14 and IKBG19 showed a moderate activity in which IKBG14 had the greatest effect on *E*. *coli*. However, the ethyl acetate extracts of all isolates had no activity towards *E. coli*. All strains except IKBG14 exhibited antibacterial activity against *P. aeruginosa*. Table 3 shows the biological activity of both extracts from endophytic isolates against the test microorganisms. The results were correlated with the bioactive compounds reported by Taechowisan, et al.^28^, Taechowisan et al.^29^. The results revealed that the isolates possess strong antibacterial activities against Gram-positive and Gram-negative bacteria, which is consistence with the findings of previous studies^17,30,31^. The results indicated that bacteria under the family of *Streptomycetaceae* presented a remarkable antagonistic activity as compared to other isolates against the test pathogens.

**Table 3:** Antibacterial activity of crude extracts obtained from endophytic actinobacteria of the medicinal plants *Thymes kotschyanus, Allium hooshidaryae*, and *Cerasus microcarpa*. The antagonist activity of extracts, in some cases, was comparatively greater than positive controls. Imipenem and vancomycin used as positive controls, and methanol and ethyl acetate were negative controls. The results were recorded as very strong inhibition (+ 4), strong inhibition (+ 3), moderate inhibition (+ 2), weak (+ 1), and no activity (−), no difference as compared to negative controls.

Isolates	Methanolic crude extract			Ethyl acetate crude extract		
	*S. aureus*	*E. coli*	*P. aeruginosa*	*S. aureus*	*E. coli*	*P. aeruginosa*
IKBG03	–	–	–	+ +	–	+ + +
IKBG05	–	–	+	–	–	–
IKBG07	+ + +	–	+ +	–	–	–
IKBG13	–	–	+	+	–	+ + +
IKBG14	+ + +	+ +	–	–	–	–
IKBG17	–	–	+ +	+	–	–
IKBG18	+	–	+ + +	+	–	+ + +
IKBG19	+ + +	+	+ +	+ +	–	+ + + +
IKBG20	–	–	+ + +	+ +	–	+ + +

## Discussion

The rising prevalence and widespread of antibiotic resistance in pathogens and cancers have hampered the efficacy of many drugs/antibiotics, and burden a heavy sustainable threat on health care system worldwide. Therefore, new strategies are required to discover novel medicines to fight against this issue. The search for novel strains from untapped environments is a smart approach to seek for new drug leads. Ample documents and evidence support that EA from untapped environments are a promising source for discovery of a wide spectrum of putative novel bioactive compounds towards antibiotic resistant pathogens. A total of nine EA was isolated and identified from three medicinal plants namely *Thymes kotschyanus, Allium hooshidaryae*, and *Cerasus microcarpa*. The majority of EA were isolated form the roots and stems. Primary identification was based on morphological characteristics (Table 1).

The color of the aerial mass, substrate mycelium and diffusible pigment were determined using the color chart from the Inter-Society Color Council–National Bureau of Standard (ISCC-NBS) color system.

The properties of mycelia and spores were also examined using light and electron scanning microscopes, and then the coloration of the colonies was determined using the ISCC-NBS system^32,33^. Among the media used for isolation, TWYE seems to be the best medium for isolation of actinobacteria, which was in line with findings of Mahdi et al.^17^ who isolated three strains using the same medium. There are many reports highlighting TWYE and HVA, which are low nutrient-media, as the best media for isolating actinobacteria^27,34,35^ in which corroborated our funding^22,23^. This is the first study to investigate the population of EA in the target plants.

The strains were observed to form abundant straight or spiral branched aerial mycelium, occasionally twisted at the end, which differentiated into straight or spiral spore chains. Scanning electron micrograph results revealed that spores of isolates were detected to be large cylindrical (IKBG03) or large sausage (IKBG05) or cylindrical (IKBG07) or small oval (IKBG13 and IKBG20) or cubed (IKBG14 and IKBG19) or small cubed (IKBG17)-shaped, with indent smooth surfaces, whereas IKBG18 had a chain of cylindrical- shaped spores with indent coarse surfaces.

The 16S rRNA gene sequence analyses revealed that the isolates belong to four families *Streptomycetaceae*, *Nocardiaceae*, *Micromonosporaceae*, and *Pseudonocardiaceae* in which *Streptomycetaceae* were predominant which is in accordance with the previous reports^17,36^. Over 50% of isolates were found to be a member of rare actinobacteria. The strains IKBG03, IKBG05, IKBG13, IKBG14 and IKBG18 belonged to rare actinobacteria. They included *Pseudonocardia* spp., *Nocardia* spp., and *Micromonospora* spp., of these, *Pseudonocardia* spp., and *Nocardia* spp., were the dominant groups. In corroboration with our results, previous studies also recorded these strains as endophytes from medicinal plants^12,37,38^. They have also been reported as main *Actinomycetes* genera in cave ecosystems^39^. All identified families are important for the production of antibiotics^36,40,41^. Numerous rare actinomycetes belonging to different genera, have been reported from cave samples as a source of antibacterial compounds during 1999 and 2018^42^. In agreement with our study, Biranvand et al. isolated and identified endophytic bacteria in a number of medicinal plants^43^. Of the 23 endophytic species isolated, seven belonged to the actinobacteria class, with families *Streptomycetaceae*, *Nocardiaceae*, *Thermomonosporaceae*, *Dietziaceae*, and *Micrococcaceae*. To the best our knowledge, to date, there is no publication on the population, and antibacterial activity of EA from *Thymes kotschyanus, Allium hooshidaryae*, and *Cerasus microcarpa*, and this is the first report. In a study of *Thymes roseus* in China, researchers have isolated a total of 126 actinobacterial species belonging to 24 genera using three methods of sample preparation of which the majority are *Streptomyces* which is consistent with previous studies^17,44^.

According to our results, there is no significant difference among the genera recovered from different segments of target plants and different genera have the same distribution. Similar to previous studies, most isolates were obtained from roots (4 isolates), followed by stems (3 isolates) and leaves (2 isolates), respectively^37,45^. In the current study, the highest diversity of genera belonged to the family *Streptomycetaceae*. This family is the largest family of actinobacteria and has been the most frequently reported genus of *Streptomyces* in the numerous literatures^31,38^.

Despite of using different strategies to maximize the isolation of endophytic actinomycetes, including diverse culture media, selection of native plants, and prolonged cultivation, up to 16 weeks, the number of isolated strains was less than expected. The reason which we have recovered a relatively lower number of isolates as compared to other studies, it might likely be due to the intrinsic feature of plant species which are herbaceous, not woody plants and are more vulnerable to the surface sterilization reagents and thus suggested that these chemicals might have penetrated to the samples and destroyed the EA or they might be unculturable on the selected media. There was also contamination of fast-growing bacteria and fungi, probably endophytic fungi, which might prevent the growth of actinobacteria and this was in line with previous publication data^27^. It seems that it is possible to enhance the number of isolation strains by media optimization, the proper time of incubation, increasing the scale of cultured samples, and further improved isolation media as well as using molecular and non-culture dependent techniques^27^.

Zhou et al. studied anti-*tuberculous* effects of YIM65484 strain, having close relationship to the IKBG19 isolate, which has high similarity to *Streptomyces cyaneofuscatus* NBRC 13062T^46^. Four antibacterial compounds were reported from YIM65484, isolated as an endophyte from *Tripterygium wilfordii*, which showed IC_50_ values of 18–64 µg/ml against *M. tuberculosis*. Based on phylogenetic analysis, the closest relative strain to IKBG07 isolate was *S. californicus* strain, which had 99.01% similarity in sequence. *S. californicus* strain ADR1 strain was isolated as an endophyte from *Datura metel*^47^. Similar to our findings, secondary metabolites of this strain exhibited substantial antibacterial activity against pathogenic bacteria such as *S. aureus*. Based on their closely related strains, IKBG03, IKBG07, IKBG20, IKBG14, and IKBG17 strains had previously been reported as endophytes^48–51^. While the IKBG05, IKBG13, and IKBG18 strains were isolated and identified as endophytes for the first time. On the other hand, IKBG03, IKBG05, IKBG13, and IKBG17, in terms of 16S rDNA, had less than 98.65% sequence similarity with their closest relative strains, for which they could be considered as new species^52^. Therefore, it is necessary to perform whole genome analyses, along with new methods such as ANI, dDDH, and MLSA to define new strains^53^. In addition, along with genomic characteristics, morphological, chemotaxonomic, and phenotypic analyses are necessary for accurate evaluation and confirmation of new species. Furthermore, the intact nature of the Iranian ecosystem along with very limited studies in this area can increase the odds of species diversity which in turn might result in discovery of new medicines. Therefore, more efforts are needed to isolate new strains with the ability to produce secondary metabolites and to study the demographic diversity of these areas.

The preliminary antibacterial activity of isolates was tested against three pathogenic bacteria namely *S*. *aureus, E. coli* and *P. aeruginosa.* Both methanolic and ethyl acetate extracts of isolates exhibited a wide range of antagonistic activities against the target pathogenic bacteria, which is in agreement with the antibacterial finding reported by Gebreyohannes et al.^54^.

Among all isolates, IKBG19, closely related strain to *S. cyaneofuscatus,* extracts exhibited a wide spectrum of antibacterial activity in comparison to other isolates against the test pathogenic bacteria. Except IKBG05, all isolates had antibacterial activity against *S.* *aureus*. Meanwhile, methanolic extracts of IKBG07, IKBG14, and IKBG19 isolates showed significant activity against *S*. *aureus*, as compared to positive control which is similar to a previous report^55^. IKBG14 and IKBG19 were also the only strains active against *E. coli*, while other isolates had no significant effect against this strain. Most of isolates exhibited a strong antibacterial activity against *P*. *aeruginosa* from which IKBG18 strain showed the highest activity. In this study, rare actinobacteria rather IKBG-14 exhibited antagonist activity against test bacteria which was consistence with finding reported by Qin et al.^56^. However, the majority of isolates showed antagonistic activities against two test pathogens. *S*. *aureus* and *P. aeruginosa* were affected by most of isolates. IKBG03, IKBG13, IKBG18, IKBG19, and IKBG20 were shown a strong growth inhibitory to *P. aeruginosa*. IKBG05, IKBG07 and IKBG17 also had moderate activity against this pathogen strain. The results of current study and other similar studies uncover that EA are still the main source of bioactive compounds that may help to discover new drugs^57,58^. Further research on identification of all secondary metabolites produced by these strains could lead to discovery of new pharmaceutical and biotechnological leads^59^. The high inhibitory activity and wide antimicrobial array of tested strains suggested that the isolated EA are potential candidates for novel antimicrobial agents.

The bioactivity of the isolates varied between *E. coli* and *P. aeruginosa*, both Gram negative bacterial strains. The results clearly indicated that *P. aeruginosa* was highly susceptible to the tested crude extracts as compared to *E. coli*. The discrepancy in sensitivity could be attributed to morphological differences between these two bacteria. For example, outer membrane of *E coli* having lipopolysaccharide which makes the cell wall impermeable to lipophilic extracts, Lipid Transfer Proteins (LTPs) and the structure of pores and efflux pumps or it might be likely due to functionally difference in the PhoP-PhoQ systems of *E. coli* and *P. aeruginosa*. Two-component systems (TCSs) assist bacteria to rapidly recognize and adapt to changes in their environment. A large number of TCSs exist in these pathogens, which are associated in regulation of gene expression in response to environmental signals such as antibiotic exposure^60^. The other example of autoregulation within TCSs is the PhoQ/PhoP system in *E. coli*. They also likely differ in the expression of periplasmic space enzymes, degrading the antibiotics. The expression of periplasmic enzymes in the intact cell relies on the presence of hydrophilic channels in the outer membrane. The outer membrane in gram-negative bacteria makes a significant role in pathogenicity of bacteria. The features of these porins are crucial for the intrinsic level of antibiotic resistance in gram-negative bacteria. The major outer membrane porin of *P. aeruginosa* (OprF) carries solutes at least two times slower than that of bacteria, such as *E. coli*^61^. *E. coli*, was generally less susceptible to antibacterial extracts which might likely be due to their outer membrane lipopolysaccharide.

## Methods

### Chemicals and reagents

All organic solvents were purchased from Merck (Darmstadt, Germany) except when the supplier was mentioned, and were either of HPLC grade or the highest degree of purity. All bacterial culture media were purchased from Merck-Millipore company (Darmstadt, Germany), Fluka (Fluka Chemie GmbH, Buchs, Switzerland), Fischer (Fischer Scientific GmbH, Schwerte, Germany), or Sigma Aldrich (Sigma Aldrich Chemie GmbH, München, Germany) unless otherwise stated. All chemicals used for DNA extraction were purchased from Sigma-Aldrich (St. Louis, MO) and SinaClon.

### Sample collection

The healthy plant specimens of *Thymes kotschyanus,* and *Allium hooshidaryae* were collected from mountainous areas bordering Iran and Iraq in Piranshahr city, West Azerbaijan province, Iran, ((36° 47′ 44′′ N 44° 58′ 47′′ E), and C*erasus microcarpa* was collected off mountainous region of Goldareh, Nojubaran, Kermanshah province, Iran (34° 25′ 46′′ N 47° 25′ 23′′ E, 1570m sea level) during the period of April 2019 to May 2020. All the procedure related to plants were complied with relevant institutional, national, and international guidelines and legislations. The collection of specimens was done randomly from the wild under the Research Center of Agriculture and Natural Resources of Kermanshah (RANK) permit, provided by the governments. The plant specimens were then identified and authenticated by experts at the RANK and Research Center of Agricultural and Natural Resources of Kurdistan Sanandj (HKS) according to the Flora of Iran and Flora Iranica. *Thymes kotschyanus* and *Allium hooshidaryae* were identified by a botanist (Mr. Hiva Ghaderi), and their voucher specimens 6639 and 8858 were then deposited in the herbarium of HKS, Iran, respectively. *Cerasus microcarpa* was identified by Dr Nastaran Jalilian, a botanist at Kermanshah Agricultural and Natural Resources Research and Education Centre, Kermanshah, Iran, and a voucher number (10,730) was deposited at the herbarium of RANK. All plant samples were packed in sterilized polythene bags and transferred to the laboratory for the surface sterilization and isolation of EA within 24 h. The plants were dissected to stems, leaves, roots, and bulbs, surface-sterilized and cultured within 48 h of sampling.

### Sample sterilization and cultivation

The surface sterilization of plant samples was conducted according to the methods described by Ali et al^31^ and Mahdi et al^17^. Briefly, the samples were first washed several times with running tap water to remove all contaminants including soil, sand, epiphytic and rhizospheric microorganisms. They were then cut into small pieces of one to two centimeters with a sterile scalpel and subjected to surface sterilization and validation as reported by^17,27^. First, the samples were immersed in 1% Tween-20 solution for 5 min, then in 70% ethanol for 5 min, followed by 5 min washing in 6% NaOCl and then washed 5 times with sterile distilled water to eliminate the chemicals. Finally, the tissues were immersed in sterile 10% NaHCO_3_ for 10 min to delay the growth of endophytic fungi and washed three times with sterile double distilled water. Upon sterilization, the samples were air-dried in a laminar air flow.

The sterilized tissues were imprinted on standard culture media namely TWYE, YECD, HVA, and PDA to isolate EA (Table 4)^37^. The pH of all media was adjusted to 7.2. The surface-sterilized samples were then aseptically dissected into 1–2 cm and placed directly on the culture media in triplicate and the petri-plates incubated at 28 ± 2 °C, and the growth of bacterial samples was monitored over 16 weeks^62^. The isolation media were supplemented with nalidixic acid (10 µg/ml) and benomyl (25 µg/ml) to inhibit the growth of gram-negative bacteria and fungi, respectively^58^. The growth of actinobacteria colonies was regularly tracked by their morphological traits^6^. By emerging of each colony, the putative isolate with the typical morphology of actinobacteria colonies was picked out from isolation media, and transferred and inoculated onto ISP2 medium and purified. Pure cultures were obtained after two to three successive sub-culturing rounds and transferred to the fresh isolation media. Colonies were purified through repeated streaking onto half-strength potato dextrose agar (HPDA, Difco) plates. The pure isolated strains were then maintained on ISP2 cultures and used as master plates to establish stock cultures and the isolates stored in Tryptic Soybean Broth medium (TSB) containing 20% glycerol at − 20°C for further analysis.

**Table 4:** Ingredient of culture media used for endophytic actinobacteria isolation.

Medium	Composition	Refs.
TWYE	Yeast extract 0.25 g, K_2_HPO_4_ 0.5 g, agar 18 g	^34^
YECD	Yeast extract 0.3 g, K_2_HPO_4_ 2.0 g, glucose 0.3 g, agar 18 g	^63^
HVA	Humic acid 1.0 g, CaCO_3_ 0.020 g, Na_2_HPO_4_ 0.5 g, KCl 1.7 g, MgSO_4_. 7H_2_O 0.05 g, FeSO_4_.7H_2_O 0.01 g, Na_2_HPO_4_ 0.5 g, agar 18 g	^64^
PDA	Potato extract 4.0 g, dextrose 20.0 g, agar 20.0 g	^65^

To ensure the efficacy of the surface sterilization, 100 μL of the last double distilled water was cultured onto ISP-2 media and incubated at 28 ± 2 °C for one week. No growth of any bacteria or fungi was considered as effective surface sterilization^17,37^.

### Morphological characterization of bacteria

The isolates were sub-cultured onto ISP2 for 10–14 days at 28 °C, and monitored daily. The color of aerial and matrix hyphae and spores was ascertained as compared with the color chart from the ISCC-NBS. Morphological characteristics of strains were studied. The colonies were classified based on their macroscopic, morphological and cultural characteristics including mycelia, the color of culture and diffusible pigments. Microscopic monitoring of stained (Gram staining) and unstained (wet mount) bacteria was further applied for identification. Morphological differences of isolates were recorded according to color of aerial and substrate mycelia, the presence or absence of spores, spore shape, spore chain morphology, pigment or melanin production of colony and mycelium color and growth characteristics after 10 days of incubation onto ISP-2 medium, following the general guidelines of the International Streptomyces Project^66,67^.

The isolates were then studied by scanning electron microscopy (SEM) (FEI, quanta 450, America) according to the methods described by Mahdi et al.^17^. The isolates were cultured onto ISP2, the aerial mycelia and spores were obtained on a coverslip. Briefly, sterile coverslips were inserted into the ISP2 solidified medium plate at an inclination of 45° with the agar surface. The isolates were inoculated along the surface of the medium which meets the surface of the buried coverslip^36^, and incubated at 28 °C for ten days. The coverslips were then removed using sterile forceps and specimens were then splutter-coated with a film of gold about 150–200 A° thickness under vacuum, and then viewed on the SEM at an accelerating voltage of 25.000–30.000 V. The mycelium and spore surface structures, spore chain morphology and ornamentation of the potential strains were analyzed.

### DNA extraction and 16S rDNA amplification

Genomic DNA extraction and PCR amplification of the 16S rDNA of strains were conducted based on Mahdi et al. and Ali et al.^17,31^. Biomass for molecular analysis was obtained by cultivation of the strains in TSB. Briefly, pure isolates, scrapped from ISP2 culture, were inoculated into TSB medium and incubated at 140 rpm in a rotary shaker (HYSC, Korea) for 7 days at 28 ± 2 °C. After growth, the mycelium was centrifugated using Hettich 320R, Germany (4000 rpm, 10 min, at 4 °C) to separate the supernatant. The biomass were then used to extract DNA or study their antimicrobial activities The Genomic DNA was extracted according to Ali et al.^17,31,34^. For each isolate, 70–100 mg of bacterial pellet was weighed and washed twice with 500 μL of Tris–EDTA (10 mM Tris, 1 mM EDTA, pH 8.0) by vortex and centrifuging (5 min, 5000 RCF). After that, 500 µL TE buffer was added to each sample and then 0.2 mg ml^−1^ of Lysozyme enzyme added to each microtube and incubated for 60 min at 37 °C. Subsequently, 10 μL of 1% (w/v) proteinase K enzyme and 10 μL of 10% sodium dodecyl sulfate was added, and the mixed solution was incubated at 55 °C for 60 min. Afterwards, 100 µL 5M NaCl and 65 µL CTAB (0.27 M) /NaCl (0.7 M) were added to the microtubes and incubated for 10 min at 55 ℃, and after a short vortex spined down. The sample was then extracted with an equal volume of chloroform-isoamyl alcohol (24:1) (600 µL) and incubated for 30 min at room temperature followed by centrifugation using Hermle Z216M, Germany (12,000 RCF, 15 min). The aqueous phase was carefully transferred to a new microtube and washed with 500 µL chloroform. After 15 min of incubation at room temperature, the lysate was centrifuged (12,000 RCF, 15 min) and the aqueous phase transferred to a new microtube followed by adding 0.4 mg mL^−1^ of RNase and incubation at 37 ℃ for 60 min. Washing with chloroform was repeated. The mixture was then treated with 1X volume of 3M sodium acetate and 3X volume of 99% cold ethanol and kept in a freezer at – 20 ℃ overnight. After centrifugation at 16,000 RCF for 15 min, the supernatant was discarded and the DNA precipitated, the pellet was then washed twice with 70% ethanol. The dried pellet was then resuspended in 50 µl of sterile H_2_O.The quality and quantity of extracted genomic DNA were checked at A260nm/280 nm and A260 nm/230 nm ratios by Nanodrop 2000 spectrophotometer (Thermo Scientific, USA), and on 1% agarose gel and stored at −20 ℃ until further use.

The isolates were subjected to 16S rDNA gene sequence analysis. The 16S rDNA was amplified using Mastercycler® nexus, Eppendorf. The 16s RNA gene of isolates was amplified using universal primers 27F (AGAGTTTGATCMTGGCTCAG) and 1492R (TACGGGGTACCTTGTTACGACTT), synthesized by Metabion, Germany^68^. Polymerase chain reaction (PCR) was carried out in a total reaction volume of 25 μL as described by Coombs and Franco^34^ consisting of 1 μL of DNA template (50–100 ng), 0.3 μL of each primer (10 pmol/μL), 1 μL of dNTPs (2.5 mM each), 2.5 μL of 1 × PCR buffer, 0.75 μL MgCl_2_ (1.5 mM), and 0.125 μL of Taq DNA polymerase (5 U/μL), and 19.025 distillated water (D.W). PCR conditions included an initial denaturation at 94 °C for two min, followed by 30 cycles of denaturation at 94 °C for one min, 50 °C for one min, and 72 °C for two min and a final extension at 72 °C for 10 min and then cooled to 4 °C. The PCR products were then separated and confirmed by gel electrophoresis and UV-transilluminator. The 1.5 Kb amplified 16S rDNA fragments was monitored on 1% agarose gel electrophoresis and visualized using a Quantum-ST4, GelDoc (France). After determining the appropriate concentration of DNA using (Nanodrop 2000 spectrophotometer, Thermo Scientific, USA), the PCR products were sequenced by Macrogen Inc., on an Abi3130 DNA sequencer (Seoul, South Korea).

### Phylogenetic analysis

The closely related strains to isolates were identified through phylogenetic analyses. The taxonomy characterization of isolates was according to 16S rDNA analysis**.** The 16S rDNA gene sequences were achieved by the BLASTn searches program and then compared with existing sequences of bacteria available in the National Center for Biotechnology Information (NCBI) and EzBioCloud databases to determine similarity percentages^69,70^. Multiple DNA sequence alignment of selected 16S rDNA was performed using the ClustalW (version 1.83) algorithm in the MEGA X software^71^. The phylogenetic tree was constructed by MEGA X using maximum- likelihood method based on the Kimura 2-parameter model^72^. Pairwise distances for the neighbor-joining algorithm were calculated based on the Kimura two-parameter model (Kimura 1980). Strength and reliability (topologies of the neighbor- joining) of the resultant tree was assessed after a 1000 bootstrap-replicate analysis^73^. *Escherichia coli* was used as an outgroup strain for tree constructions^74^. The 16S rDNA gene sequences of the isolates were then deposited in GenBank and their accession numbers acquired. The phylogenetic tree was constructed using the neighbor-joining algorithm/method (based on 1000 bootstrap iterations) of nucleotides sequence of 16S rDNA gene.

### Preparation of crude extract for antibacterial evaluation

The pure isolates were grown on TSA medium (7 days, 28 ± 2 °C). The full plate containing the culture and the agar medium was then cut into small pieces with a sterile scalpel, and placed into 250 ml Erlenmeyer flasks containing 50 mL of ethyl acetate, and put on a shaker for 24h (200 RPM) at room temperature. The ethyl acetate solution was filtered and the supernatant collected. Then, 50 mL of methanol was added into the flasks, allowing the device to operate for another 24 h under the same conditions, the extract was separated. Both extracts were concentrated under reduced pressure using a rotary evaporator (70 RPM, 38°C) to yield a dry extract. Then, the extracts were stored at – 20 °C until used for evaluation of antimicrobial activity.

### Evaluation of antibacterial activity

The antibacterial potential of the extracts was evaluated against *Staphylococcus aureus* ATCC 25923, *Escherichia coli* ATCC 25922 and *Pseudomonas aeruginosa* ATCC 27853, using a typical agar diffusion assay^75^. The test organisms were grown in tryptone soy broth (TSB, Merck) and incubated at 37 °C for 18–24 h. The growth of the cultures was evaluated by measuring the optical density (OD) using a Spekol 1500, spectrophotometer at 600 nm (OD_600 nm_), and OD was adjusted to 0.2. The antibiotic agar medium No.1 (AAM- MERCK) was seeded with the test culture (1% V/V) and dispensed into petri dish plates, and 6 mm diameter wells were prepared using a sterile cork borer at regular intervals to make 10 wells. Each well was then filled with 50 μL of extracts, allowing the wells to dry fully, and the plates were incubated at 37 °C for 24h. Vancomycin (25 µg/ml) and Imipenem (30 µg/ml *P. aeruginosa* ATCC 27853 and 1.5 µg/ml *E. coli* ATCC 25922) were used as positive controls for Gram-positive and Gram-negative bacteria, respectively, with ethyl acetate and methanol as negative controls. Each test was conducted in duplicates and repeated twice. The diameters of the zone of inhibition around each well were then compared with the negative and positive controls.

## Conclusion, future prospectives and directions

Antibiotic resistance and nosocomial infections are a global health problem and one of the major causes of death among hospitalized patients. Therefore; to fight against the bacterial resistance, it is a clear need to develop new antibiotics, particularly against Gram-negative bacteria which have some superior features such as a second polar outer membrane and numerous efflux pumps compared to Gram-positive bacteria that render these bacteria less susceptible to antibiotic treatments. Natural products have generally had crucial roles in improving human health and wellbeing, namely their application in microbial remedies and cancer prevention and treatment regimens. Our study revealed that plants are rich sources for the isolation and identification of EA with a potential to produce a vast array of bioactive metabolites which have a wide range of pharmaceutical and biotechnological applications. This study, for the first time, the plants *Thymes kotschyanus, Allium hooshidaryae*, and *Cerasus microcarpa* were selected to identify the population of EA. A total of nine EA was isolated and identified from different tissues of these plants. Most isolates gave different pigmentation. The well-developed, branched substrate hyphae and aerial mycelia differentiate into straight or spiral spore chains composed of cylindrical or cube or oval spores with smooth or indented or rough surfaces. While *Streptomyces* was the most prevalent genus among isolates and have a cosmopolitan distribution, several isolates that show bioactivity belonged to rare actinomycetes. The data corroborated the results reported by^44^ who isolated a number of *Streptomyces* from *Thymus roseus*. Based on 16S rDNA sequence and phylogenetic analyses, the IKBG03, IKBG05 IKBG13, and IKBG17 isolates could be considered as potentially novel species; therefore, more genomic studies and biosynthetic gene clusters analyses of the active strains and polyphasic study into novel strains are needed. 

To increase the number of identities, it seems that media with low levels of nutrients are more suitable to isolate actinobacteria and result in the isolation of a large number of actinobacteria or it might use the unculturable methods such as molecular techniques for identification of isolates to increase the number.

All strains exhibited substantial antibacterial effects against at least one of the test pathogens, indicating the potential of these strains to produce secondary metabolites. This study provides a valuable snapshot of the antibacterial potentials of EA from untapped native plants in Iran, having activity against both Gram-positive and Gram-negative bacteria. To the best of our knowledge, this is the first report on antibacterial property of novel strains from these plants. The IKBG18 and IKBG20 strains showed a potent antibacterial activity. 

This research topic expands our knowledge on the actinobacteria- derived bioactive compounds and their biological potential applications. We may need to use different production media to induce silent cluster genes as previous studies highlighted the importance of media for production of bioactive compounds^75^. Further research is necessary to evaluate the novelty of molecules responsible for the preliminary bioactivity. Further chemical study is also required to purify and elucidate the structure of bioactive compounds from extracts with antibacterial activity in order to develop putative drugs against resistant bacteria. Previous reports have insinuated that the derived actinomycetes bioactive compounds are of alkaloids, macrolides, phenolic, diketopiperazine (DKP), peptides, polyketides, xiamycins, dilactones, salinosporamides, indole alkaloids, 2-pyranones, sesquiterpenes, and phenazines^7,76^. Therefore, it is vital to perform bioassay-guided fractionation and purification researches on the extracts to explore the structure of pure compounds. Besides, the mechanism of action of bioactive compounds must be scrutinized before they can be used as therapeutic agents. 

### Supplementary Information

Supplementary Figure 1.

Supplementary Figure 2.

Supplementary Figure 3.

## Data Availability

The nucleotide sequences generated during the current study have been deposited in the GenBank/EMBL/DDBJ databases under the following accession numbers: MZ733426, MZ733428, MZ733430, MZ733436, MZ733440, MZ733437, MZ733441, MZ580433 and MZ820169.
