# Supplementary material for: Identification and anti-bacterial property of endophytic actinobacteria from Thymes kotschyanus, Allium hooshidaryae, and Cerasus microcarpa
Source: Sci Rep. 2023 Aug 12;13:13145. doi: 10.1038/s41598-023-40478-x (PMC10423286; doi:10.1038/s41598-023-40478-x)
Supplement: Supplementary file 1 — Supplementary Figure 1. [file 41598_2023_40478_MOESM1_ESM.docx]

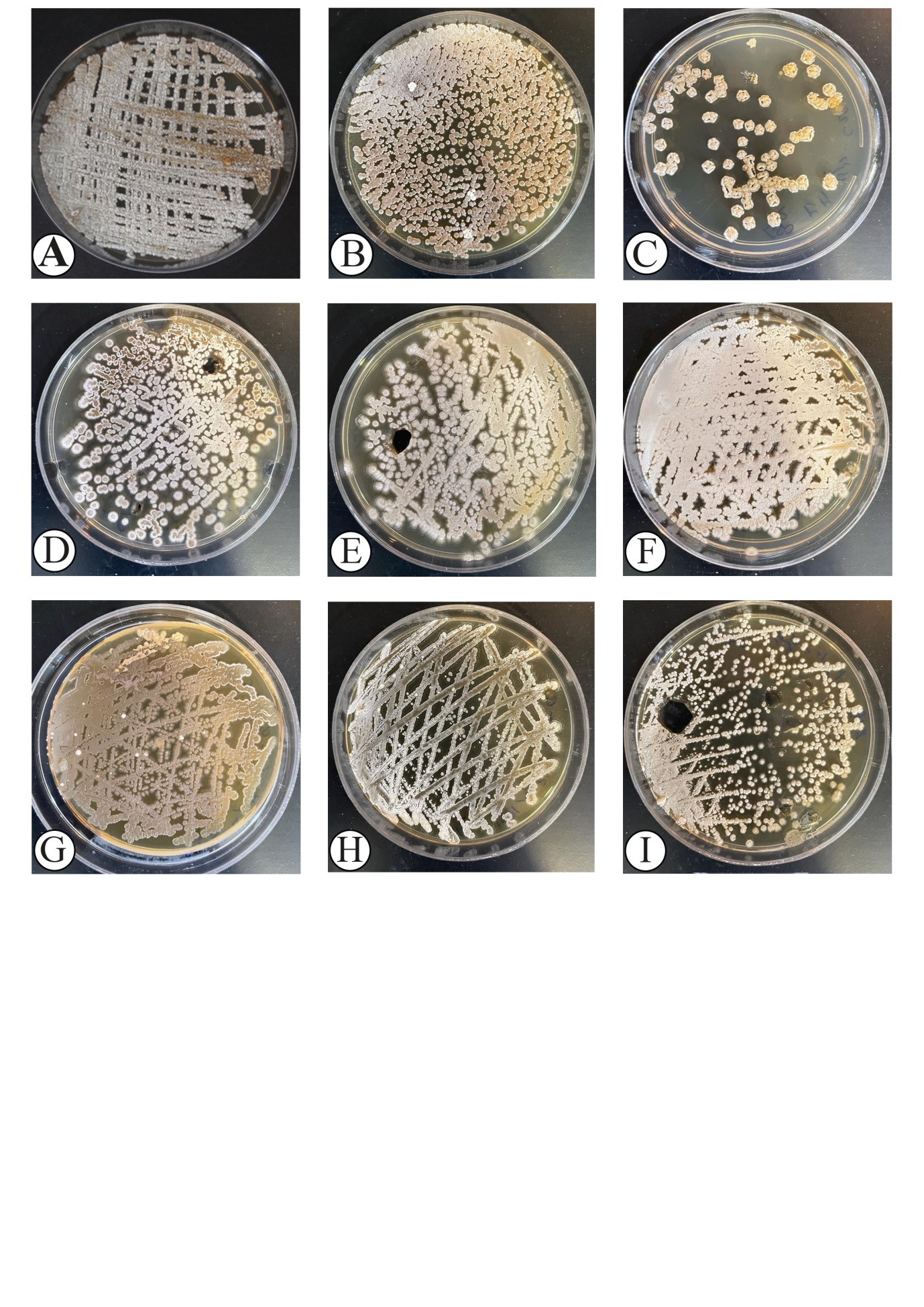


**Supplementary Figure 1;** Morphological characterization of actinobacteria isolates**.**

Colony morphology of actinobacteria isolates after two weeks of incubation onto ISP2 at 28^o^C. IKBG03 (A), IKBG05 (B), IKBG07 (C), IKBG13 (D), IKBG14 (E), IKBG17 (F), IKBG18 (G), IKBG19 (H), IKBG20 (I).
