# Supplementary material for: Identification and anti-bacterial property of endophytic actinobacteria from Thymes kotschyanus, Allium hooshidaryae, and Cerasus microcarpa
Source: Sci Rep. 2023 Aug 12;13:13145. doi: 10.1038/s41598-023-40478-x (PMC10423286; doi:10.1038/s41598-023-40478-x)
Supplement: Supplementary file 2 — Supplementary Figure 2. [file 41598_2023_40478_MOESM2_ESM.docx]

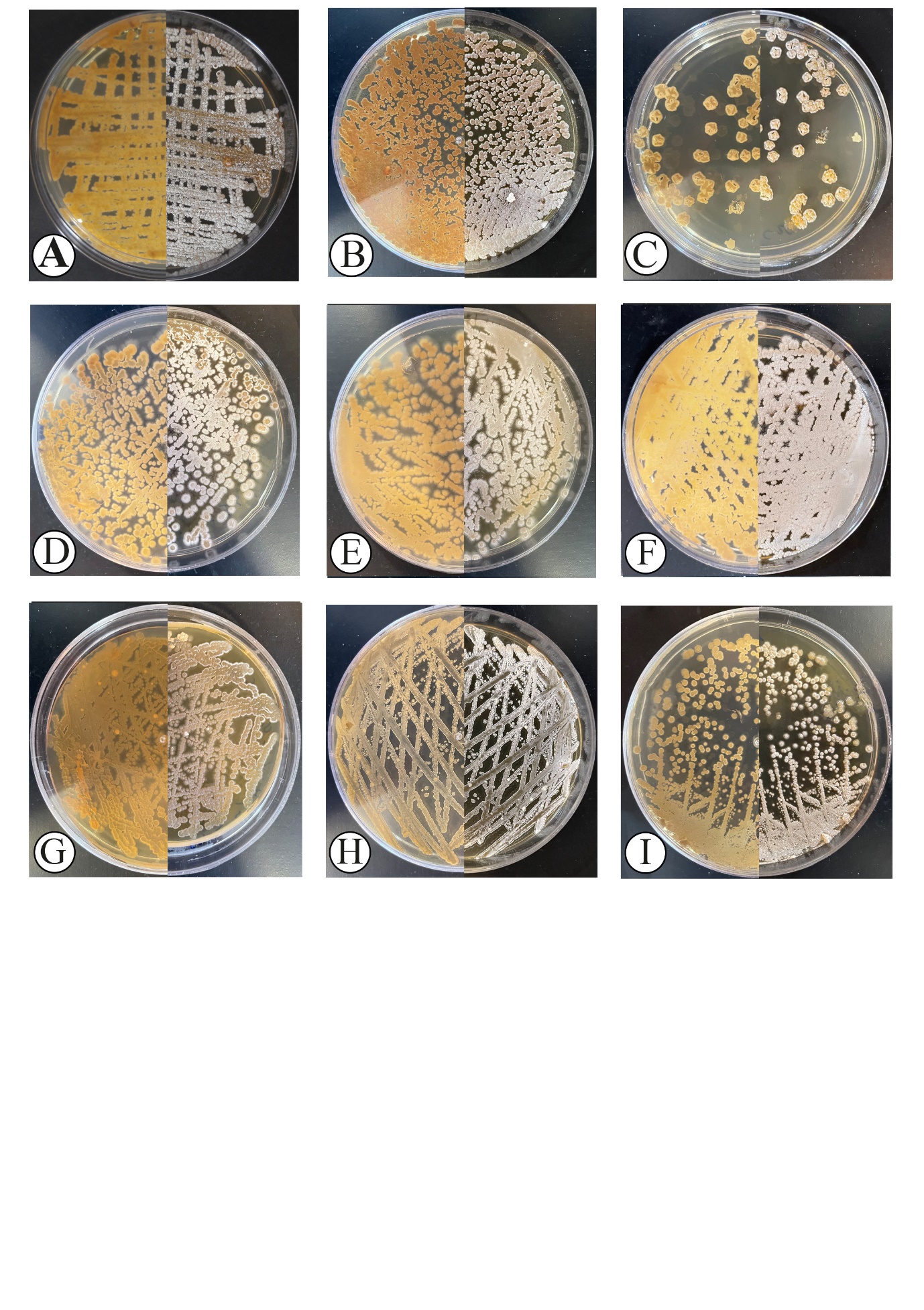


**Supplementary Figure 2;** Morphological characterization of actinobacteria isolates**.**

Colony morphology of actinobacteria isolates after two weeks of incubation onto ISP2 at 28^o^C. IKBG03 (A), IKBG05 (B), IKBG07 (C), IKBG13 (D), IKBG14 (E), IKBG17 (F), IKBG18 (G), IKBG19 (H), IKBG20 (I). The right half of each plate shows the front of culture, while the left half shows the back of each culture.
