# Supplementary material for: Identification and anti-bacterial property of endophytic actinobacteria from Thymes kotschyanus, Allium hooshidaryae, and Cerasus microcarpa
Source: Sci Rep. 2023 Aug 12;13:13145. doi: 10.1038/s41598-023-40478-x (PMC10423286; doi:10.1038/s41598-023-40478-x)
Supplement: Supplementary file 3 — Supplementary Figure 3. [file 41598_2023_40478_MOESM3_ESM.docx]

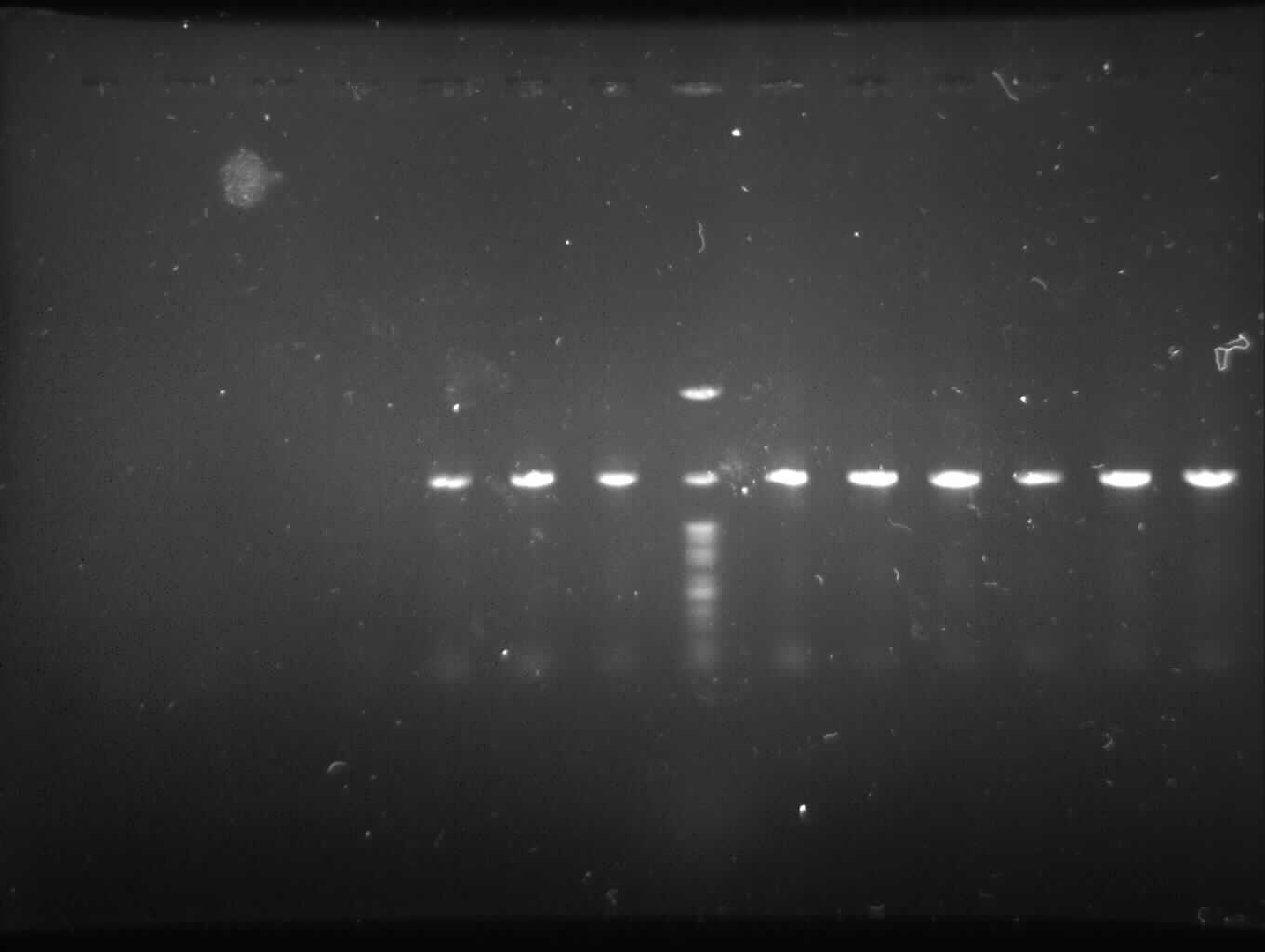


**Supplementary Figure 3;** The original image of PCR products gel**.**

Gel electrophoresis of PCR products. PCR products of 16S rDNA of isolated strains using 27F and 1492R primers that amplify a 1.5 Kb product from strains. The 1.5 Kb amplified 16S rDNA fragments observed on 1% agarose gel electrophoresis. From left to right; IKBG03 (lane 1), IKBG05 (lane 2), IKBG07 (lane 3), DNA ladder (lane 4, SinaClon, Iran), IKBG13 (lane 5), IKBG14 (lane 6), IKBG17 (lane 7), IKBG18 (lane 8), IKBG19 (lane 9), IKBG20 (lane 10).
